# Feasibility and acceptability of human papillomavirus self-sampling compared with clinician sampling in urban areas of western China: a cross-sectional survey

**DOI:** 10.3389/fpubh.2025.1524796

**Published:** 2025-04-23

**Authors:** Shaolong Xue, Xi Zeng, Jing Li, Leni Kang, Mingrong Xi, Lian Xu, Ping Fu, Min Zhou, Mengyin Ao, Xiaoxi Yao, Dongmei Li, Guangdong Liao

**Affiliations:** ^1^Department of Gynecology, West China Second University Hospital, Sichuan University, Chengdu, China; ^2^Key Laboratory of Birth Defects and Related Diseases of Women and Children, Ministry of Education, Sichuan University, Chengdu, China; ^3^West China School of Public Health and West China Fourth Hospital, Sichuan University, Chengdu, China; ^4^National Office for Maternal and Child Health Surveillance, West China Second University Hospital, Sichuan University, Chengdu, China; ^5^Department of Pathology, West China Second University Hospital, Sichuan University, Chengdu, China; ^6^Chengdu Shuangliu District Maternity and Child Healthcare Hospital, Chengdu, China

**Keywords:** vaginal self-sampling, urine self-sampling, human papillomavirus testing, cervical intraepithelial neoplasia, cervical cancer screening, acceptability

## Abstract

**Introduction:**

Cervical cancer, driven by persistent high-risk human papillomavirus (hrHPV) infection, remains a global health challenge, especially in low- and middle-income areas such as western China. Despite the critical role of HPV testing in early detection, coverage in China remains low due to cultural, psychological, and other barriers. Self-collected urine and vaginal samples offer alternative methods for sample collection. This study aimed to evaluate the feasibility and acceptability of detecting hrHPV and cervical intraepithelial neoplasia grade 2 or worse (CIN2+) via urine and vaginal self-sampling compared with clinician sampling in urban areas of western China.

**Methods:**

A cross-sectional survey was conducted from November 2022 to March 2023 in urban areas of western China. The participants provided self-collected urine and vaginal samples for hrHPV testing and completed questionnaires on acceptability of self-sampling. The HPV positivity, agreement, and kappa value were calculated to assess concordance between self- and clinician sampling. The sensitivity, specificity, agreement, predictive values, and likelihood ratios were used to evaluate the clinical performance of both methods for detecting CIN2+.

**Results:**

A total of 2,228 female subjects aged 21–71 years were recruited, and self-collected urine samples, vaginal samples, and clinician-collected cervical samples were obtained. The sensitivity of clinician sampling, urine self-sampling and vaginal self-sampling were 80.00% (95% CI: 44.22–96.46), 70.00% (95% CI: 35.37–91.91) and 90.00% (95% CI: 54.12–99.48) for CIN2+; the specificity for <CIN2 were 98.33% (95% CI: 97.68–98.81), 98.23% (95% CI: 97.56–98.72) and 98.50% (95% CI: 97.87–98.95%); and the agreements for CIN2+ were 98.25% (95% CI: 97.59–98.74), 98.83 (95% CI: 98.26–99.22) and 98.82 (95% CI: 98.25–99.21). All methods yielded high negative predictive values, high positive likelihood ratios, and low negative likelihood ratios. Additionally, participants reported high acceptability of self-sampling, citing less discomfort and embarrassment than clinician sampling.

**Conclusion:**

Self-collected urine and vaginal samples for the detection of hrHPV and CIN2+ demonstrate high diagnostic accuracy and acceptability, making them viable alternatives to clinician-collected samples. Self-sampling methods may improve screening accessibility and compliance, especially in resource-limited settings, thereby supporting the prevention and early detection of CIN2+.

## 1 Introduction

Cervical cancer, which is strongly associated with persistent high-risk human papillomavirus (hrHPV) infection, ranks as the fourth most common cancer in both incidence and mortality among women, with approximately 660,000 new cases and 350,000 deaths globally in 2022 (1). Notably, 80% of the decline in cases occurred in lower-middle-income countries (LMICs) (2). Unfortunately, China remains among the developing countries with a high burden of cervical cancer, making it the most prevalent gynecological cancer in terms of both incidence and mortality (3). Cervical cancer could be the first cancer eliminated worldwide through strategies such as the 90–70–90 targets proposed by the World Health Organization (WHO) in 2020. These targets aim for 90% of girls to be fully vaccinated with the HPV vaccine by age 15, 70% of women to be screened with a high-performance test by age 35 and again by age 45, and 90% of women diagnosed with precancer or invasive cancer to receive appropriate treatment (4).

In addition to vaccination, HPV testing is a crucial method for cervical cancer screening, offering greater effectiveness in reducing cervical cancer incidence compared to cervical cytology, due to its higher sensitivity and negative predictive value (5–8). As of 2021, 48 countries have recommended primary HPV-focused screening, either independently or in combination with other screening methods such as cytology (9). Low rates of HPV vaccination and cervical cancer screening coverage remain significant challenges in China, where the cumulative full vaccination rate among women aged 9–45 was only 6.21% (10), and 70.5% of women aged 20–64 years have never been screened (11). This situation is attributed to organizational barriers such as limited medical access and prolonged wait times; interpersonal challenges such as stigma and a lack of family support; and personal factors such as low risk perception, discomfort, and fear (12, 13).

HPV self-sampling techniques are recommended by the WHO to be incorporated into cervical cancer screening protocols, aiming to facilitate the achievement of the 2030 targets (4, 14). As of 2021, 17 countries have integrated self-sampling into national programs or guidelines, targeting underscreened populations or as a primary method. Several studies have shown the high feasibility and acceptability of the HPV-DNA test using self-collected urine/vaginal samples and clinician-collected cervical samples (13, 15–18). Advantages include being less invasive, offering greater accessibility for self-collection, and reducing the need for infrastructure such as examination beds and costly collection kits. Self-sampling methods have also been shown to be cost-effective (19–21), making them a promising way to increase women’s participation in cervical cancer prevention, especially in LMICs.

Despite evidence supporting the feasibility and acceptability of self-sampling methods, studies in China on these techniques remain limited (22, 23). Therefore, our study aimed to evaluate the feasibility and acceptability of detecting high-risk HPV (hrHPV) and cervical intraepithelial neoplasia grade 2 or worse (CIN2+) using urine and vaginal self-sampling compared with clinician sampling in urban areas of western China.

## 2 Methods

### 2.1 Study design and participants

A cross-sectional study was conducted from November 2022 to March 2023 in urban areas of western China. To be eligible for the study, women had to be between 21 and 71 years of age at the time of invitation, must have refrained from sexual intercourse, vaginal medication, vaginal contraceptives, and the use of vaginal cleansers for at least 48 h prior to the examination, and must have signed the informed consent. Patients were excluded from the study if they had a history of uterine or cervical surgery (such as hysterectomy), were pregnant or within 8 weeks postpartum, or were experiencing menstrual bleeding at the time of the examination.

Eligible patients were recruited at our partner hospital in Shuangliu District, Chengdu, where they registered, signed informed consent, underwent cervical and self-sampling, and completed a questionnaire. The physician-collected hrHPV-DNA test was performed at the partner hospital, while the self-sampling hrHPV-DNA test was conducted at Hangzhou central laboratory. Cytology and immunohistochemistry were handled by Guangzhou central laboratory and sent to our hospital’s pathology department for slide review. Colposcopy and sampling were performed at our partner hospital, with slides prepared and reviewed by our hospital’s pathology department.

### 2.2 Basic information collection, sample collection and acceptability assessment

After registration and informed consent, the participants completed a questionnaire covered basic participant information (such as age, education, marriage, family income), histories of cervical cancer prevention, previous female reproductive system diseases, menstrual cycles, and contraceptive use.

Before sample collection, video guidance based on the manufacturer’s instructions for self-sampling was given to all participants. The participants were instructed to collect at least 25 ccs of anterior first void in a sterile urine container. After urine collection, a self-sampling swab for vaginal sampling was obtained. Researchers remained outside the bathroom to offer further assistance and collect the samples. Finally, trained gynecologists conducted gynecological examinations and collected cervical samples, completing a corresponding examination questionnaire.

After sampling, the participants completed a questionnaire assessing the acceptability of the HPV screening protocol. This survey contained 20 questions evaluating their experiences, comfort, and safety during the procedure, as well as their method preferences and reasons for those choices. All paper questionnaires were transcribed into a Microsoft Access 2016 database by two data stewards for data cleaning and coding, with a third steward ensuring consistency of the double-entered data.

### 2.3 hrHPV-DNA detection, cytology, immunocytochemistry, colposcopy, and histopathology

Cervical samples were evaluated for hrHPV-DNA with HBRT-H14 (Hybribio Biotechnology Ltd. Corp., Guangdong, China) (24), whereas urine and vaginal self-samples were evaluated with CareHPV systems (QIAGEN, Hilden, Germany).

Liquid-based cytology testing (LCT) and P16/Ki-67 immunocytochemistry (ICC) were conducted on clinician-collected cervical samples if urine, vaginal, or cervical samples tested negative for HPV16/18 but positive for the other 12 types of hrHPV.

According to the screening results, women meeting any of the following criteria are required to undergo colposcopy within 1 month: (1) positive for HPV16/18; (2) positive for the other 12 types of hrHPV, along with a positive result for LCT [atypical squamous cells of undetermined significance (ASC-US) or higher] or P16/Ki-67 ICC. Tissue biopsies were performed by an experienced physician on women who tested positive during colposcopy. If abnormalities were detected, a direct biopsy was taken at the abnormal site. If a high-grade lesion was indicated by cytology but no abnormalities were found during colposcopy, endocervical curettage (ECC) and random biopsies (at positions 2, 4, 8, and 10 on the cervical squamocolumnar junction) were conducted.

All LCT, ICC, and histopathology samples were blinded and independently evaluated by two experienced cytopathologists or histopathologists. In cases of diagnostic discrepancies, the specimens were re-evaluated to reach a consensus. Cytological diagnoses were made according to the Bethesda System (3rd Ed.), and the histological results were based on the highest level of diagnosis.

### 2.4 Statistical analysis

Statistical analyses were conducted via SPSS version 23.0 (IBM Corp., Armonk, NY, United States) and VassarStats (online). The HPV positivity rate, agreement rate, and kappa value were calculated to assess the concordance between self-sampling and clinician sampling. The sensitivity, specificity, agreement rate, predictive values, and likelihood ratios were used to evaluate the clinical performance of self-sampling and clinician sampling for detecting CIN2+. HPV positivity rates between self-sampling and physician sampling were compared using McNemar’s test. Participants’ feelings about self-sampling were compared using Chi-Square test, and Bonferroni’s correction was used for multiple comparisons. The distributions of difficulty levels between self-sampling and physician sampling were compared using the Mann–Whitney U test. All tests were two-sided, and *p* values < 0.05 were considered statistically significant.

### 2.5 Ethics approval and consent to participate

This study was approved by the ethics committees of both the Peking Union Medical College Hospital (PUMCH) (approval code ZS-3293) and West China Second University Hospital (WCSUH). Written informed consent from all the study participants was obtained, and all materials complied with the Declaration of Helsinki and ethical standards.

## 3 Results

**Figure 1:**
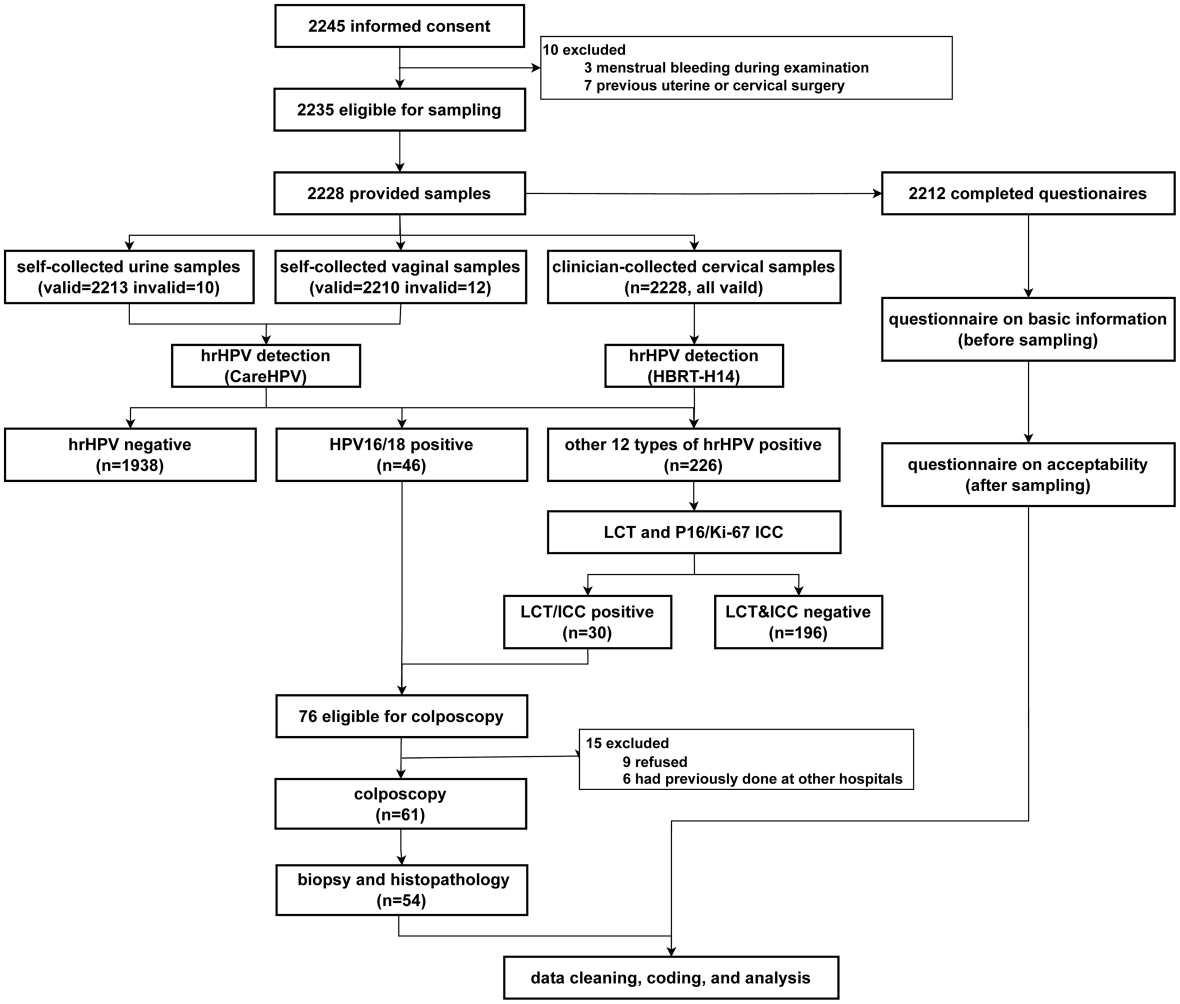
Study flowchart.

Figure 1 presents a flow chart of the study. Among the 2,235 eligible participants, 2,228 provided self-collected urine and vaginal samples, and 2,212 completed the questionnaire.

### 3.1 Basic characteristics

The median age of the 2,228 participants was 52 years (IQR: 40–64 years). Most participants were married (2,071/2,228, 93.0%), and approximately 98.8% (2,202/2,228) had completed primary or higher education. Additionally, 91.4% (2,037/2,228) had not received the HPV vaccine, and 65.5% (1,459/2,228) had never undergone cervical screening (Table 1).

**Table 1:** Characteristics of the participants (*N* = 2,228).

Characteristics	No. of women *n*, %
Age [median (IQR)]	52 (40–64)
Education	
College graduate or more	599 (26.9)
High school graduate	377 (16.9)
Middle school graduate	925 (41.5)
Primary graduate	301 (13.5)
Uneducated	26 (1.2)
Marriage	
Married/living with partner	2,071 (93.0)
Divorced/separated/widowed	131 (5.9)
Single	26 (1.2)
Contraception (within 3 years)	
Yes	680 (30.5)
No	1,548 (69.5)
HPV vaccination	
Yes	191 (8.6)
No	2,037 (91.4)
Previous cervical screening	
Yes	769 (34.5)
No	1,459 (65.5)
Cervical screening time	
Within 3 years	620 (80.6)
Beyond 3 years	131 (17.0)
Not sure	18 (2.3)
Cervical screening method	
Pap smear or LCT	516 (67.1)
HPV testing	405 (52.7)
VIA/VILI	11 (1.4)
Other	6 (0.8)
Not sure	121 (15.7)

The results are presented as *n* (%) if not otherwise stated.

IQR, interquartile range; HPV, human papillomavirus; LCT, liquid-based cytology test; VIA/VILI, visual inspection with acetic acid and Lugol’s iodine.

### 3.2 Accordance of HPV testing between self-sampling and clinician sampling

The comparison among the tests is shown in Table 2 and Supplementary Table 1. The hrHPV positivity rate was similar between urine and clinician samples (9.9% vs. 9.9%, *p* > 0.05) and between vaginal and clinician samples (9.4% vs. 9.9%, *p* > 0.05).

**Table 2:** Results of hrHPV testing—self-collected urine and vaginal samples compared with clinician-collected cervical samples.

Method	TP	TN	FP	FN	McNemar’s χ^2^	*p* value	OPA, % (95% CI)	PPA, % (95% CI)	NPA, % (95% CI)	Cohen’s Kappa (95% CI)
hrHPV										
Urine self-sampling	172	1,945	49	47	0.04	> 0.05	95.66 (94.71 ~ 96.46)	78.54 (72.39 ~ 83.66)	97.54 (96.74 ~ 98.16)	0.758 (0.711 ~ 0.804)
Vaginal self-sampling	176	1,959	32	43	1.61	> 0.05	96.61 (95.74 ~ 97.30)	80.37 (74.35 ~ 85.29)	98.39 (97.71 ~ 98.88)	0.806 (0.763 ~ 0.848)
Combined self-sampling^a^	186	1,927	54	33	5.07	< 0.05	96.05 (95.12 ~ 96.80)	84.93 (79.34 ~ 89.26)	97.27 (96.43 ~ 97.93)	0.788 (0.746 ~ 0.831)
HPV 16/18^b^										
Urine self-sampling	30	2,170	10	3	2.77	> 0.05	99.41 (98.97 ~ 99.67)	90.91 (74.53 ~ 97.62)	99.54 (99.13 ~ 99.77)	0.819 (0.722 ~ 0.916)
Vaginal self-sampling	29	2,170	7	4	0.36	> 0.05	99.50 (99.08 ~ 99.74)	87.88 (70.86 ~ 96.04)	99.68 (99.31 ~ 99.86)	0.838 (0.744 ~ 0.932)
Combined self-sampling^a^	31	2,154	13	2	6.67	< 0.05	99.32 (98.85 ~ 99.60)	93.94 (78.38 ~ 98.94)	99.40 (98.95 ~ 99.67)	0.802 (0.704 ~ 0.901)
Other 12 types of hrHPV										
Urine self-sampling	146	1,974	46	47	0.01	> 0.05	95.80 (94.85 ~ 96.58)	75.65 (68.86 ~ 81.40)	97.72 (96.95 ~ 98.31)	0.735 (0.684 ~ 0.787)
Vaginal self-sampling	152	1,990	27	41	2.88	> 0.05	96.92 (96.09 ~ 97.59)	78.76 (72.18 ~ 84.17)	98.66 (98.03 ~ 99.10)	0.800 (0.754 ~ 0.846)
Combined self-sampling^a^	159	1,958	49	34	2.71	> 0.05	96.23 (95.32 ~ 96.97)	82.38 (76.11 ~ 87.33)	97.56 (96.76 ~ 98.17)	0.772 (0.725 ~ 0.820)

hrHPV, high-risk human papillomavirus; TP, true positive; TN, true negative; FP, false positive; FN, false negative; OPA, overall percent agreement; PPA, positive percent agreement; NPA, negative percent agreement; CI, confidence interval.

a Either sample (urine or vaginal sample) tested HPV positive is considered as positive.

b Either HPV 16 or HPV 18 positive is considered as positive.

Urine self-sampling had an overall percent agreement (OPA) of 95.66%, a positive percent agreement (PPA) of 78.54%, and a negative percent agreement (NPA) of 97.54%, with a Cohen’s kappa of 0.758. Vaginal self-sampling had an OPA of 96.61%, a PPA of 80.37%, and a NPA of 98.39%, with a Cohen’s kappa of 0.806. The combined self-sampling methods yielded an OPA of 96.05%, a PPA of 84.93%, and a NPA of 97.27%, with a Cohen’s kappa of 0.788.

### 3.3 Cytological and immunocytochemical findings of the population

Among those with cytological alterations (25/226, 10.8%), ASC-US was the most common (20/226, 8.6%). ICC was positive for both P16 and Ki-67 in 17 samples (7.5%). Overall, 30 samples (13.3%) were positive for either cytology (ASC-US or higher) or ICC [P16 (+), Ki-67 (+)] (Supplementary Table 2).

### 3.4 Histological findings of the population

Of the 76 participants eligible for colposcopy, 61 (80.2%) completed the procedure, with 54 cervical samples (71.1%) collected. Histological findings showed 24 women (44.4%) with less than cervical intraepithelial neoplasia grade 1 (< CIN1), 20 (37.0%) with CIN1, and 10 (18.5%) with grade 2 or worse (≥ CIN2) (Supplementary Table 3).

The accuracy rates for detecting CIN2+ lesions are shown in Table 3. Clinician-collected cervical samples had a sensitivity of 80.00% and a specificity of 98.33%, and an agreement of 98.25%. Urine self-sampling had a lower sensitivity of 70.00% and a specificity of 98.23%, and an agreement of 98.10%. Vaginal self-sampling had a sensitivity of 90.00% and a specificity of 98.50%, and an agreement of 98.46%. The combined approach of urine and vaginal self-sampling maintained a sensitivity of 90.00% and a specificity of 98.17%, with an agreement of 98.14%. All methods yielded high negative predictive values (NPVs), high positive likelihood ratios (PLRs), and low negative likelihood ratios (NLRs).

**Table 3:** Accuracy rates for the detection of CIN2+ in cervical, vaginal, and urine samples.

Method	TP	TN	FP	FN	Sensitivity, % (95% CI)	Specificity, % (95% CI)	Agreement, % (95% CI)	PPV, % (95% CI)	NPV, % (95% CI)	PLR (95% CI)	NLR (95% CI)
Clinician routine^a^	8	2,181	37	2	80.00 (44.22 ~ 96.46)	98.33 (97.68 ~ 98.81)	98.25 (97.59 ~ 98.74)	17.78 (8.51 ~ 32.58)	99.91 (99.63 ~ 99.98)	47.96 (30.73 ~ 74.84)	0.20 (0.06 ~ 0.70)
Urine routine^b^	7	2,164	39	3	70.00 (35.37 ~ 91.91)	98.23 (97.56 ~ 98.72)	98.10 (97.42 ~ 98.61)	15.22 (6.84 ~ 29.48)	99.86 (99.56 ~ 99.96)	39.54 (23.71 ~ 65.93)	0.31 (0.12 ~ 0.79)
Vaginal routine^b^	9	2,167	33	1	90.00 (54.12 ~ 99.48)	98.50 (97.87 ~ 98.95)	98.46 (97.83 ~ 98.92)	21.43 (10.84 ~ 37.24)	99.95 (99.70 ~ 100)	60.00 (40.35 ~ 89.21)	0.10 (0.02 ~ 0.65)
Combined routine^c^	9	2,150	40	1	90.00 (54.12 ~ 99.48)	98.17 (97.50 ~ 98.68)	98.14 (97.46 ~ 98.64)	18.37 (9.24 ~ 32.50)	99.95 (99.70 ~ 100)	49.28 (34.03 ~ 71.34)	0.10 (0.02 ~ 0.65)

HPV: Human papillomavirus; TP: true positive; TN: true negative; FP: false positive; FN: false negative; PPV: positive predictive value; NPV: negative predictive value; PLR: positive likelihood ratio; NLR: negative likelihood ratio; CI: confidence interval.

a Defined as HPV16/18 positive in clinician-collected samples, or positive for other hrHPV types in clinician-collected samples with concurrent positive LCT/ICC.

b Defined as HPV16/18 positive in self-collected urine/vaginal samples, or positive for other hrHPV types in self-collected urine/vaginal samples with concurrent positive LCT/ICC.

c Either routine (urine or vaginal routine) positive is considered as positive.

### 3.5 Awareness, feelings, and acceptability of self-sampling

The participants’ awareness of cervical cancer, HPV, and self-sampling is presented in Table 4. Awareness of cervical cancer was high, with 86.1% (1,905/2,212) of participants aware of the disease, and 71.6% (1,583/2,212) believing it could be prevented. In contrast, only 35% (775/2,212) were aware of HPV, and 88.9% (245/2,212) had never heard of HPV self-sampling. Among those aware of HPV self-sampling, medical professionals were the primary source of information (51.4%, 126/245).

**Table 4:** Participants’ awareness of cervical cancer, HPV, and self-sampling (*n* = 2,212).

Awareness	No. of women *n*, %	
Are you aware of cervical cancer?	*Yes*—1,905 (86.1)	*No*—307 (13.9)
Do you think cervical cancer can be prevented?	*Yes*—1,583 (71.6)	*No*—64 (2.9)
	*Not sure*—565 (25.5)	
Are you aware of human papillomavirus or HPV?	*Yes*—775 (35.0)	*No*—1,437 (65.0)
Are you aware of HPV self-sampling?	*Yes*—245 (11.1)	*No*—1,967 (88.9)
How did you learn about HPV self-sampling?		
Participated in research projects.	33 (13.5)	
By hospital or medical professionals.	126 (51.4)	
Heard from relatives or friends.	45 (18.4)	
Seen online.	46 (18.8)	

The results are presented as *n* (%). HPV, human papillomavirus.

The participants’ feelings about self-sampling are presented in Table 5. Most were confident in performing self-sampling (urine vs. vaginal: 97.7% vs. 86.5%, *p* < 0.0001). Embarrassment was reported less frequently during urine self-sampling (3.6%, 80/2,212) than during vaginal (12.5%, 277/2,212, *p* < 0.0001) or clinician-collected sampling (11.8%, 262/2,212, *p* < 0.0001). Discomfort or pain was reported less frequently during urine self-sampling (2.2%, 48/2,212) than during vaginal (18.6%, 412/2,212, *p* < 0.0001) or clinician-collected sampling (48.4%, 1,071/2,212, *p* < 0.0001), and less frequently during vaginal self-sampling than during clinician-collected sampling (18.6% vs. 48.4%, *p* < 0.0001).

**Table 5:** Participants’ feelings about self-sampling (*n* = 2,212).

Feelings		No. of women *n*, %			Pearson χ^2^	*p* value^a^
		Urine self-sampling	Vaginal self-sampling	Clinician sampling		
I was able to collect samples correctly.	Yes	2,162 (97.7)	1,914 (86.5)	**–**		
	No	46 (2.1)	292 (13.2)	**–**	194.13	< 0.0001
	Refusal^b^	4 (0.2)	6 (0.3)	**–**		
I felt embarrassed during sampling.	Yes	80 (3.6)	277 (12.5)	262 (11.8)	U vs. C: 105.51	< 0.0001
	No	2,111 (95.4)	1,916 (86.6)	1,924 (87.0)	U vs. V: 118.15	< 0.0001
	Refusal^b^	21 (0.9)	19 (0.9)	26 (1.2)	V vs. C: 0.42	> 0.017
I felt discomfort or pain during sampling.	Yes	48 (2.2)	412 (18.6)	1,071 (48.4)	U vs. C: 1257.08	< 0.0001
	No	2,141 (96.8)	1,771 (80.1)	1,117 (50.5)	U vs. V: 323.02	< 0.0001
	Refusal^b^	23 (1.0)	29 (1.3)	24 (1.1)	V vs. C: 440.94	< 0.0001

The results are presented as *n* (%).

a Bonferroni’s correction for multiple comparisons. *p* values < 0.017 were considered statistically significant.

b The participant refused to answer this question.

U: urine self-sampling; V: vaginal self-sampling; C: clinician-collected sampling.

The participants’ preferences for self-sampling are presented in Table 6. Most (75.9%, *n* = 1,679) favored clinician sampling, mainly because of trust in physicians (94.2%, *n* = 1,582) and concerns about incorrect sampling (34.1%, *n* = 573). In contrast, 22.0% (*n* = 487) preferred self-sampling, citing the inconvenience of gynecological exams (39.8%, *n* = 194), better privacy (37.2%, *n* = 181), and less pain and fear (33.5%, *n* = 163). Among those preferring clinician sampling, 41.6% (*n* = 699) would choose self-sampling if clinician sampling was unavailable. When comparing urine to vaginal self-sampling, 35.5% (*n* = 785) preferred urine for its convenience (96.3%, *n* = 756), 11.0% (*n* = 243) preferred vaginal self-sampling for its reliability (94.2%, *n* = 229), 52.8% (*n* = 1,167) had no strong preference.

**Table 6:** Participants’ preferences for self-sampling (*n* = 2,212).

Preference	No. of women *n*, %	
Which do you prefer: self-sampling or clinician-collected sampling? Why?		
Clinician sampling	1,679 (75.9)	
Trust in physicians.	1,582 (94.2)	
Lack of confidence in self-sampling.	573 (34.1)	
Fear of injury during self-sampling.	93 (5.5)	
Other reasons.	31 (1.8)	
Self-sampling	487 (22.1)	
Gynecological examinations are inconvenient.	194 (39.8)	
Transportation to the hospital is inconvenient.	65 (13.3)	
Better protection of privacy.	181 (37.2)	
Less pain and fear.	163 (33.5)	
Other reasons.	126 (25.9)	
Not sure or refuse to answer	46 (2.1)	
(For participants who prefer clinician-collected sampling) If clinician-collected sampling were not available, would you choose self-sampling?	Yes—699 (41.6)	Maybe—153 (9.1)
	No—775 (46.2)	Not sure—61 (3.6)
Which do you prefer: urine self-sampling or vaginal self-sampling? Why?		
Urine self-sampling	785 (35.5)	
More convenient.	756 (96.3)	
Vaginal self-sampling may hurt oneself.	54 (6.9)	
Vaginal self-sampling equipment may not clean.	30 (3.8)	
Other reasons.	13 (1.7)	
Vaginal self-sampling	243 (11.0)	
More reliable.	229 (94.2)	
More convenient.	24 (9.9)	
Other reasons.	27 (11.1)	
Both	1,167 (52.8)	
Not sure or refuse to answer	17 (0.8)	

The results are presented as *n* (%).

The participants’ acceptance of self-sampling is presented in Table 7. Urine self-sampling was perceived as easier than vaginal self-sampling (Z = −34.0, *p* < 0.001). Urine sampling was rated as “very easy” by 72.7% (*n* = 1,608) and “relatively easy” by 25.9% (*n* = 572), while for vaginal sampling, the rating were 30.4% (*n* = 672) and 46.4% (*n* = 1,026). Regarding preferred locations for self-sampling, 61.7% (*n* = 1,365) preferred guidance by medical staff in hospitals. Additionally, 86.2% (*n* = 1,907) were willing to send self-collected samples to a hospital or testing institution, and 80.9% (*n* = 1,790) were willing to recommend self-sampling to others.

**Table 7:** Participants’ acceptance of self-sampling (*n* = 2,212).

Acceptance	No. of women *n*, %	
The difficulty level of urine self-sampling		
1—very easy	1,608 (72.7)	
2—relatively easy	572 (25.9)	
3—somewhat difficult	24 (1.1)	
4—very difficult	2 (0.1)	
Difficult to describe or refuse to answer	6 (0.3)	Mann–Whitney U test Z: −34.0 P < 0.001
The difficulty level of vaginal self-sampling		
1—very easy	672 (30.4)	
2—relatively easy	1,026 (46.4)	
3—somewhat difficult	419 (18.9)	
4—very difficult	61 (2.8)	
Difficult to describe or refuse to answer	34 (1.5)	
Place for self-sampling		
At home.	337 (15.2)	
A private room in the hospital.	188 (8.5)	
Guided by medical staff.	1,365 (61.7)	
All the above.	272 (12.3)	
Not sure.	42 (1.9)	
Refuse to answer	8 (0.4)	
Would you be willing to send the self-collected samples to the hospital or testing institution?	Yes—1,907 (86.2)	No—183 (8.3)
	Not sure—199 (5.4)	Refuse to answer—3 (0.1)
Would you be willing to recommend self-sampling technology to others?	Yes—1,790 (80.9)	No—274 (12.4)
	Not sure—138 (6.2)	Refuse to answer—10 (0.5)

The results are presented as *n* (%).

## 4 Discussion

This study aimed to evaluate the feasibility and acceptability of detecting hrHPV and CIN2+ via urine and vaginal self-sampling compared with clinician sampling in urban areas of western China.

Our study evaluated the feasibility and acceptability of self-sampling in a population with low vaccination and screening rates. The HPV vaccination rate among the study population was 8.6% (191/2,228), which is higher than the national average in China (10) but lower than the global average of 15% (25). This may be because Shuangliu, the study location, was among the first regions in China to initiate cervical cancer vaccination programs. The overall cervical cancer screening rate was 34.5% (769/2,228), which was lower than the national average in China of 36.8% (95% CI: 35.1–38.4) (11).

Self-sampling could be a viable alternative in hrHPV screening, potentially improving accessibility and compliance. Arbyn’s 2022 meta-analysis (16) of 4 studies conducted in screening populations reported a pooled OPA between vaginal self-sampling and clinician sampling of 88.1% (95% CI: 81.8–93.3), a kappa statistic of 0.65 (95% CI: 0.51–0.79), a PPA of 74.8% (95% CI: 60.0–87.1), and a NPA of 92.0% (95% CI: 86.9–94.8). Bober’s 2021 meta-analysis (26) reported the diagnostic accuracy of first-void urine sampling versus clinician-collected sampling. For hrHPV detection, the sensitivity of urine self-sampling was 78% (95% CI: 70–84), and the specificity was 89% (95% CI: 81–94). For HPV16/18 detection, the sensitivity of urine self-sampling was 87% (95% CI: 74–94), and the specificity was 91% (95% CI: 83–96). Our study demonstrated strong concordance between urine and vaginal self-sampling and clinician-collected samples for hrHPV detection, with kappa values of 0.758 for urine, 0.806 for vaginal, and 0.788 for the combined methods. All three self-sampling methods (urine, vaginal and combined) resulted in high OPA, PPA, and NPA.

Our findings concerning the detection of CIN2+ are consistent with those of other studies investigating the accuracy of HPV testing via self-collected vaginal or urine samples. Previous studies, including clinical trials and meta-analyses, have shown that HPV testing on self-collected vaginal or urine samples has a similar accuracy to that of clinician-collected cervical samples (15, 27–32). For example, a 2018 meta-analysis by Arbyn et al. (27) revealed that compared with clinician sampling, PCR-based hrHPV assays had similar diagnostic accuracy for CIN2+ or CIN3+ patients via vaginal self-sampling (pooled sensitivity ratio for CIN2+: 99, 95% CI: 0.97–1.02). Additionally, two randomized trials (28, 32) confirmed comparable accuracy between self-collected and clinician-collected samples for detecting CIN2+ or CIN3+ lesions. The 2021 meta-analysis by Cho et al. (33) demonstrated that HPV testing via a PCR-based urine detection method showed similar clinical accuracy to clinician-collected samples in detecting CIN2 or more severe lesions.

In terms of participants’ awareness of cervical cancer, HPV and self-sampling, the majority were aware of cervical cancer and thought that it could be prevented. However, their knowledge of HPV is limited and may contribute to the low rates of HPV vaccination and cervical cancer screening in China. Most participants (88.9%, *n* = 1,967) were introduced to self-sampling techniques and underwent self-sampling for the first time throughout our project. Consequently, their experiences in this self-sampling initiative could directly influence their acceptance of the technique. Since self-sampling is not yet widely adopted for cervical cancer screening in China, our study offers a genuine reflection of initial impressions and acceptance levels among the screening population. Consistent with previous studies (34, 35), most participants were confident in their ability to collect samples correctly (97.7%, *n* = 2,162) and reported less embarrassment (3.6%, *n* = 80) and pain (2.2%, *n* = 48) during the process.

Nelson’s 2017 meta-analysis (36) evaluated patient acceptance and preferences for self-sampling versus clinician-collected sampling across 23 studies (N = 12,610), finding an average preference rate of 59% (95% CI: 0.48–0.69) for self-sampling. In our study, only 22.1% (*n* = 487) of the participants preferred self-sampling, whereas most (75.9%, *n* = 1,679) favored clinician sampling because of their trust in physicians (94.2%, *n* = 1,582), lack of confidence in self-sampling (34.1%, *n* = 573), and fear of self-inflicted injury (5.5%, *n* = 93). This may be due to low awareness of self-sampling and the fact that most participants were sampling themselves for the first time. However, if clinician assistance was unavailable, half (50.7%, *n* = 852) of those who preferred clinician sampling would opt for self-sampling. When asked about their preferred self-sampling method, most (50.7%, *n* = 852) reported that both urine and vaginal self-sampling were acceptable. Those favoring urine sampling cited convenience (96.3%, *n* = 756), whereas those preferring vaginal sampling considered it more reliable (94.2%, *n* = 229). Thus, in areas with limited medical resources, self-sampling could be a viable alternative to clinician-collected sampling. Urine self-sampling was considered easier (Mann–Whitney U test, Z: −34.0, *p* < 0.001), suggesting that it may achieve greater acceptance in resource-limited settings.

Nelson’s 2017 meta-analysis (36) reported that 97% (95% CI: 0.95–0.98) of patients reported that self-sampling was generally acceptable (7 studies, N = 1,470), and 87% (95% CI: 0.73–0.95) expressed a willingness to repeat it (9 studies, N = 2,660). In our study, 86.2% (*n* = 1,907) of the participants were willing to send self-collected samples to a hospital or testing institution, and 80.9% (*n* = 1,790) were willing to introduce self-sampling to others, indicating a promising outlook for promoting self-sampling. Di Gennaro’s meta-analysis (13) revealed similar preferences for home sampling (66, 95% CI: 57–74%) and clinical settings (67, 95% CI: 62–71%; *p* = 0.841), whereas Nishimura’s review (37) of 8 studies revealed a preference for home collection. In contrast, our study, which provided video and professional instructions, revealed that most participants (61.7%, *n* = 1,365) preferred self-sampling under medical guidance. This preference may stem from a lack of confidence in self-sampling skills and concerns about accessing treatment when needed. These findings suggest that combining media with professional guidance could effectively promote self-sampling adoption.

Our study presents certain innovations. It focuses on western China, where low- to middle-income populations are more common and where medical resources, screening rates, and compliance are lower than those in the more developed eastern regions. Given the limited research in this area, our study on the feasibility and acceptability of self-sampling provides valuable insights and potential strategies to improve screening rates and compliance in this region.

However, our study has several limitations. As this study focused on primary screening, the low number of CIN2+ cases may have affected the feasibility assessment. Expanding the screening scale is necessary to ensure a more representative evaluation of diagnostic accuracy. Self-sampling techniques, especially urine self-sampling, need further refinement to increase sensitivity and specificity. Additionally, multicenter, large-scale prospective studies are needed to provide higher-level evidence for the adoption of HPV self-sampling.

## 5 Conclusion

Self-collected urine and vaginal samples for the detection of hrHPV and CIN2+ demonstrate high diagnostic accuracy and acceptability, making them viable alternatives to clinician-collected samples. Self-sampling methods may improve screening accessibility and compliance, especially in resource-limited settings, thereby supporting the prevention and early detection of CIN2+.

## Data Availability

The raw data supporting the conclusions of this article will be made available by the authors, without undue reservation.
