# Supplementary material for: Feasibility and acceptability of human papillomavirus self-sampling compared with clinician sampling in urban areas of western China: a cross-sectional survey
Source: Front Public Health. 2025 Apr 23;13:1524796. doi: 10.3389/fpubh.2025.1524796 (PMC12055758; doi:10.3389/fpubh.2025.1524796)
Supplement: Supplementary file 1 [file Data_Sheet_1.docx]

**Supplementary Table 1 Positivity of high-risk HPV for clinician-collected and self-collected samples in the study population.**

| **Method** | **Total** | **Invalid** | **hrHPV** | **Other 12 hrHPV** | **HPV16/18** |
| --- | --- | --- | --- | --- | --- |
| Clinician-collected samples | 2228 | 0 | 219 (9.83) | 193 (8.66) | 35 (1.57) |
| Urine samples | 2223 | 10 (0.45) | 221 (9.94) | 192 (8.64) | 42 (1.89) |
| Vaginal samples | 2222 | 12 (0.54) | 208 (9.36) | 179 (8.06) | 38 (1.71) |

Results are presented as n (%).

hrHPV: high-risk human papillomavirus.

**Supplementary Table 2.** **Cytological and immunocytochemical characteristics of samples positive only for other 12 types of high-risk HPV.**

| **LCT/ICC result** | **N (%) 226 (100 %)** | **N (% over the total) hrHPV positive** | | | |
| --- | --- | --- | --- | --- | --- |
|  |  | **Clinician sampling**  **N=180** | **Urine self-sampling**  **N=181** | **Vaginal self-sampling N=170** | **Combined self-sampling^a^ N=196** |
| **LCT** | | | | | |
| NILM | 202 (89.4) | 159 (78.7) | 161 (79.7) | 150 (74.3) | 175 (86.6) |
| ≥ ASC-US | 23 (10.2) | 21 (91.3) | 19 (82.6) | 19 (82.6) | 20 (87.0) |
| ≥ LSIL | 5 (2.2) | 5 (100) | 5 (100) | 5 (100) | 5 (100) |
| Not valuable^b^ | 1 (0.4) | 0 | 1 (100) | 1 (100) | 1 (100) |
| **ICC** |  |  |  |  |  |
| P16(-) Ki-67(-) | 209 (92.5) | 164 (78.5) | 167 (79.9) | 157 (75.1) | 181 (86.6) |
| P16(+) Ki-67(+) | 17 (7.5) | 16 (94.1) | 14 (82.4) | 13 (76.5) | 15 (88.2) |
| Either positive^c^ | 30 (13.3) | 28 (93.3) | 24 (80.0) | 23 (76.7) | 26 (86.7) |

ICC: immunocytochemistry; hrHPV: high-risk human papillomavirus; NILM: negative for intraepithelial lesion or malignancy; ASC-US: atypical squamous cell of undetermined significance; ASC-H: atypical squamous cells - cannot exclude high grade squamous intraepithelial lesion; LSIL: low-grade squamous intraepithelial lesion. HSIL: high-grade intraepithelial lesion. AIS: adenocarcinoma in situ.
a Either sample (urine or vaginal sample) tested positive other 12 types of hrHPV was considered as positive.

b Not valuable: Not able to classify the samples due to insufficient material or artifacts.

c Either positive: a positive result of Cytology (ASC-US or higher) or ICC (P16(+) Ki-67(+)).

**Supplementary Table 3. HPV-positive cervical, vaginal, and urine samples according to histology.**

| Histopathology Results | N Total (%) 54 (100 %) | **N (% over the total) hrHPV positive** | | | |
| --- | --- | --- | --- | --- | --- |
|  |  | **Clinician sampling**  **N=48** | **Urine self-sampling**  **N=46** | **Vaginal self-sampling**  **N=43** | **Combined self-sampling^a^  N=49** |
| <CIN1 | 24 (44.4) | 24 (100.0) | 21 (87.5) | 19 (79.2) | 22 (91.7) |
| CIN1 | 20 (37.0) | 16 (80.0) | 18 (90.0) | 15 (75.0) | 18 (90.0) |
| CIN2+ | 10 (18.5) | 8 (80.0) | 7 (70.0) | 9 (90.0) | 9 (90.0) |

hrHPV: high-risk human papillomavirus; CIN: cervical intraepithelial neoplasia.

a Either sample (urine or vaginal sample) tested positive other 12 types of hrHPV was considered as positive.
